# Effect of Laparoscopic Sleeve Gastrectomy on HbA1C Level in Children with Type 2 Diabetes Mellitus

**DOI:** 10.3390/medicina58070959

**Published:** 2022-07-20

**Authors:** Ashwag Asiri, Faris Alzahrani, Hashim Alghamdi, Zainab Alamri

**Affiliations:** 1 Child Health Department, College of Medicine, King Khalid University, Abha 641, Saudi Arabia; haalghamdi2@moh.gov.sa; 2 Research Center for Advanced Materials Science, King Khalid University, P.O. Box 9004, Abha 61413, Saudi Arabia; 3 The Joint Program of Postgraduate Studies in Public Health and Preventive Medicine, Abha 61421, Saudi Arabia; 4 Ministry of Health, Abha 62523, Saudi Arabia; z.alamri4@hotmail.com

**Keywords:** laparoscopic sleeve gastrectomy, obesity, type 2 diabetes, HbA1C, RBS

## Abstract

*Background and Objectives*: A third of the American adult population is currently pre-diabetic/morbidly obese and is, therefore, at an elevated risk for developing type 2 diabetes. Unfortunately, such a condition does not spare children from also developing morbid obesity, where incidence rates of childhood obesity—coupled with type 2 diabetes—are markedly elevated. Laparoscopic sleeve gastrectomy (LSG) is gradually becoming the novel benchmark in bariatric surgery for the treatment of morbid obesity and associated co-morbidities, also within pediatric cases. However, no comprehensive study has been conducted in children that emphasizes the effect of LSG on HbA1C levels within such a patient population suffering from type 2 diabetes. Aim: Since HbA1C is a major biomarker for type 2 diabetes progression, this study aimed to identify any dysregulated serum levels for this key molecular player (together with other parameters), for post-surgical monitoring of the beneficial metabolic effects of LSG surgery on type 2 diabetes amelioration/remission within pediatric patients. *Materials and Methods*: A total of 64 pediatric patients, ranging in age from 5 to 14 years old, were enrolled in this retrospective study. Multiple laboratory-based analyses datasets were also collected from individual study participants, including HbA1C and random blood sugar (RBS). All participating patients were designated for undergoing laparoscopic sleeve gastrectomy, as per standardized surgical protocols and each participant was followed-up for two years post-surgery. Laboratory investigations were re-performed in order to identify any major variations in clinical parameters. *Results*: HbA1c was significantly reduced among children, from 6.0 ± 0.8 (pre-LSG) to 5.4 ± 0.4 post-surgery, with a reduction rate of 10.9% (*p* = 0.001). Furthermore, RBS significantly decreased from 102.9 ± 34.0 (pre-LSG) to 87.1 ± 17.3 post- surgery, with a reduction rate of 15.4% (*p* = 0.036). *Conclusions*: This study provides further concrete evidence for the beneficial metabolic influence provided by LSG surgery on morbidly obese, childhood-aged patient populations, with effectiveness in reducing co-morbidity progress, in the form of type 2 diabetes, through the reduction in HbA1c levels within such patients post-surgery.

## 1. Introduction

It is a well-established fact that, due to major technological advances in modern societies in the past 50 years, the current COVID-19 pandemic and the consequent increase in sedentary lifestyles, prevalence of morbid obesity and its medical complications have increased across global population demographics at an exorbitant rate [1,2,3]. A recent report by the American Centers for Disease Control and Prevention (CDC) has also highlighted the alarming statistic that approximately 33% of the American adult population is currently pre-diabetic and is, therefore, at an elevated risk for developing type 2 diabetes and also cardiovascular conditions, with such risks being aggravated by increased body mass index (BMI) within individuals [4,5]. Unfortunately, such a condition does not spare children from developing morbid obesity, where incidence rates of childhood obesity within most developed nations are markedly elevated and carries with it the risk of the individual children incurring multiple psychological (such as reduced self-esteem and depression) and physical co-morbidities, including type 2 diabetes [6,7,8]. A recent study performed across 300 children under the age of 18 and residing in the Aseer province of the kingdom of Saudi Arabia revealed that over 50% of the investigated children had a BMI that indicated overweight or obese status [6].

Classical therapeutic measures for mitigating further increases in BMI within children includes radical shifts in dietary intake patterns, together with a drastic increase in physical activity. However, the risk of the individual obese child to fall into non-compliance for dietary/lifestyle changes is typically high. In addition, within specific cases of extremely elevated BMI and risk of incurring type 2 diabetes, such pediatric obese patients are deemed as unsuitable to be treated solely with conventional methodologies for weight loss, thus requiring more drastic additional modalities for successful obesity management.

Recently, the American Diabetes Association has approved bariatric surgery for the management of type 2 diabetes [9]. Such a surgical procedure was found to be highly beneficial in promoting even up to 20 Kg in weight loss within a span of six months and also improving overall type 2 diabetes.

Diabetes management within such patients at the post-surgical phase, as reflected in several such studies proves this procedure to be more effective than traditional methods for achieving type 2 diabetes remission [9,10,11,12,13,14]. An additional case in point would be the meta-analysis carried out by Buchwald and colleagues in 2009, investigating 621 studies involving different types of bariatric surgeries, which revealed that a vast majority of patients that underwent weight-loss following these procedures also experienced an improvement in the clinical and laboratory manifestations of their diabetic conditions [10].

In brief, bariatric surgery involves generalized gastric resection and consequent size reduction, in order to regulate exorbitant dietary-intake volumes of food that is typically synonymous with morbidly obese patient lifestyles, and was pioneered by Kennedy and colleagues in 1947 [15]. Presently, there exists at least six differing bariatric surgical procedures, although the Roux-en-Y gastric bypass procedure was found to have elevated success in achieving post-surgical type 2 diabetes remissions within such morbidly obese patient populations [13,16]. However, an emerging novel bariatric surgical technique, known as laparoscopic sleeve gastrectomy (LSG), is gradually becoming the novel benchmark in bariatric surgery for the treatment of morbid obesity and associated co-morbidities across developed nations [17]. One of the major advantages of such a bariatric surgical procedure, apart from the minimized invasiveness of the actual surgical procedure that still confers restrictive properties for dietary intake volumes, is the long-term metabolic influences exerted by this surgical technique mainly through hormonal level shifts, such as serum ghrelin levels, glucagon-like-peptide-1 (GLP-1), together with glucose-independent peptide (GIP) [18]. Such hormones have major roles within the development of type 2 diabetes. Ghrelin consists of an orexigenic peptide with the function of antagonizing leptin activities, whereby the latter is involved in metabolizing adipose tissue [19]. GLP-1 is indirectly involved in type 2 diabetes, since its binding with relevant cellular receptors affects hemoglobin A1C (HbA1C) blood levels, a key molecular player in type 2 diabetes [20]. Similarly, GIP binding onto its relevant cellular receptors has important influence over weight regulation within diabetic patients [21].

Excellent results have been presented in terms of excess weight loss and improvement of comorbidities linked to obesity in the short and medium-term follow-up, following LSG interventions. Such benefits include a rapid resolution or improvement of type 2 diabetes within 65–85% of patients, identified during short-term post-surgical follow-ups, even if a duodenojejunal shunt is typically not created during this procedure [22]. However, the detailed mechanisms by which LSG surgical procedures affect such hormonal levels within patients remain unclear, although this correlation certainly renders LSG surgery as a restrictive and additionally a metabolic therapeutic measure for inducing weight loss and regulation of type 2 diabetes in such patients.

Consequently, following such promising results for LSG surgical techniques within the morbidly obese adult population, an emerging trend is now focusing on the implementation of LSG for adolescents and children suffering from extreme cases of morbid obesity. Two recent reports did describe the beneficial effects of LSG use within a young patient population [23,24]. However, no comprehensive study has been conducted in children that emphasizes the effect of LSG onHbA1C levels within such a patient population suffering from type 2 diabetes, particularly since HbA1C is a major biomarker for type 2 diabetes progression and any dysregulated serum level for this key molecular player would be crucial for monitoring post-surgical benefit/s of LSG surgical procedure in childhood-aged patient populations.

In essence, the aim of this specific retrospective study was to determine the effect of LSG on the HbA1C levels in diabetic obese children admitted to our medical institute, with defined objectives, namely, the determination of HbA1C levels within these pediatric patients prior and post-LSG surgical procedure execution. The dataset outcomes for this study include local statistics for morbidly obese children in our institute’s care and the crystallized picture regarding the effect that LSG has over type 2 diabetes remission levels within such pediatric cases. The study conclusions will certainly shed further light onto the multiple metabolic influences provided by LSG-based bariatric surgery within such a niche patient population suffering from morbid obesity.

## 2. Methodology

### 2.1. Patient Pre-Operative Medical Data Profiling

We included participants with type 2 diabetes and no other comorbidities. A total of 64 pediatric patients, ranging in age from 5 to 14 years old, were enrolled in this retrospective study. Written, informed consent forms by the parents/guardian for each individual patient were collected prior to proceeding with this study. In addition, this study was approved by the Ethics Committee of our medical institute. Each pediatric patient had an individual data sheet containing essential information, such as age, gender, height, weight, body mass index (BMI). In addition, associated symptoms to morbid obesity were recorded for each patient, including shortness of breath, obstructive sleep apnea, joint pain/s and intolerance of exercise. Furthermore, other medical issues for each pediatric patient about to undergo LSG surgery were recorded, such as bronchial asthma, diabetes mellitus, hypertension, thyroid disease, dyslipidemia, gall stones, hepatic steatosis, or other conditions. Finally, preoperative recording of dietary and exercise programs undergone by each patient was also performed, including details such as number of program attempts and duration/program.

Multiple laboratory-based analyses datasets were also collected from the individual study participants, namely HbA1C and random blood sugar (RBS) were recorded. All laboratory analyses were performed pre-operatively for LSG, as is routine for obesity evaluation. Such analyses were additionally performed post-LSG surgery in order to identify any possibly major variations within any such parameter. All pre-/post-surgical laboratory investigations were performed in-house in an effort to minimize error introductions.

### 2.2. Surgical Procedure

All participating patients were designated for undergoing laparoscopic sleeve gastrectomy, as per standardized surgical protocols.

### 2.3. Post-LSG Patient Follow-Ups

Following the LSG surgical procedure, each individual pediatric study participant was followed-up for up to a period of two years post-surgery. Laboratory investigations were re-performed in order to identify any major variations in clinical parameters, in particular regarding HbA1c and RBS blood levels.

### 2.4. Data Analysis

All statistical analyses were performed through SPSS version 22.0^®^ (SPSS, Inc.™, Chicago, IL, USA) using two tailed tests. *p* values less than 0.05 were deemed to be statistically significant. Descriptive analysis, based on frequency and percent distribution, was performed for pediatric participant gender. HbA1c and RBS were displayed as mean with standard deviation and median. All study parameters were comparatively analyzed prior and post-LSG surgery, with percentage variations recorded for each parameter. Paired t-tests were performed to assess the significance of the reported variations across all parameters. In addition, gender-segregated HbA1c post-surgical variations were graphically analyzed.

## 3. Results

Overall, 64 pediatric patients were enrolled in this study. All study participants were diagnosed with morbid obesity and type 2 diabetes and consequently underwent LSG surgery. The patients’ ages ranged from 5 to 14 years old, with the mean age being 11.2 ± 2.3 years old. The female cohort consisted of 34 (53.1%) participants, while male participants totaled 30 (46.9%) (Table 1).

**Figure 1:**
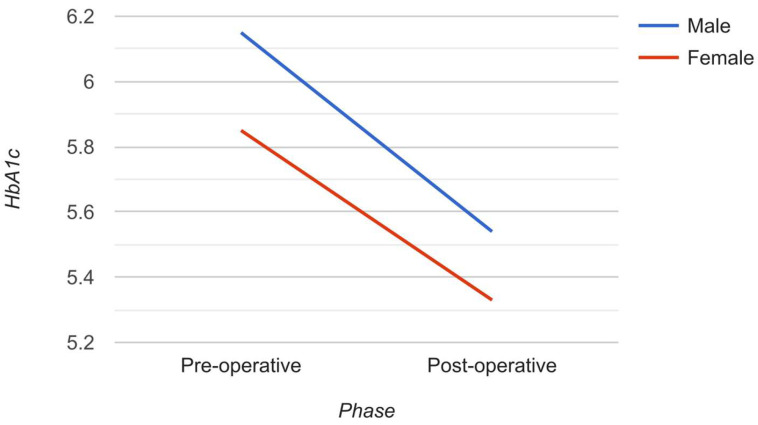
Variations in HbA1c level prior and post-LSG surgery, by child gender. Both male and female pediatric participants showed significant reduction in Hb1Ac levels following LSG surgery, with nearly equivalent reduction rates (−11% and −9.7%, respectively; *p* = 0.002).

HbA1c was found to be markedly reduced within pediatric participants, from 6.0 ± 0.8 prior to LSG surgery to 5.4 ± 0.4 post-surgery, with a reduction rate of 10.9% (*p* = 0.001). In addition, RBS was also markedly reduced, from 102.9 ± 34.0 prior to surgery to 87.1 ± 17.3 post-surgery, with a reduction rate of 15.4% (*p* = 0.036) (Table 2). Both male and female participants demonstrated marked reduction in Hb1Ac levels post-LSG, with almost equivalent reduction rates (−11% and −9.7%, respectively; *p* = 0.002) (Figure 1).

HbA1c and RBS variations among study participants prior/post-laparoscopic sleeve gastrectomy. HbA1c significantly decreased among children, from 6.0 ± 0.8 before undergoing LSG, to 5.4 ± 0.4 post-surgery, with a reduction rate of 10.9% (*p* = 0.001). Furthermore, RBS significantly decreases from 102.9 ± 34.0 prior to surgery, to 87.1 ± 17.3 post-surgery, with a reduction rate of 15.4% (*p* = 0.036, Table 2).

An ANCOVA was run to determine the effect of age on post-operative HbA1c after controlling for pre-operative HbA1c. After adjustment for pre-operative HbA1c, there was not a statistically significant difference in post-operative HbA1c between the age groups, F (2, 60) = 0.470, *p* = 0.627 (Table 3)

## 4. Discussion

The benefits of LSG surgical techniques as a therapeutic measure for obtaining obesity-driven weight loss and also as a metabolic-driven regulation of type 2 diabetes is presently established within the adult patient population suffering from morbid obesity. However, formal and comprehensive studies on the efficacy of LSG surgical procedures for achieving such goals therapeutic goals within the adolescent and childhood-age patient population are still lacking.

Presently, only two detailed reports on the use of LSG within adolescents have been described within scientific literature repositories. The study conducted by Michalsky and colleagues in 2018 focused mainly on the effect of adolescent bariatric surgical procedures (Roux-en-Y/vertical sleeve gastrectomy) on 242 adolescents admitted to 5 separate medical centers [23]. In particular, the aim of this study was on monitoring shifts in cardiovascular factors from the period prior to surgery until three years post-surgery [23]. The parameters monitored included triglycerides, lipoprotein levels, glucose, insulin and also HbA1c levels, among others. Regarding HbA1c, a modest reduction of 0.3% was observed from the pre-surgical analysis (5.4%) to three-year postsurgical analysis (5.1%) [23]. A similar study, conducted by El-Matbouly and colleagues in the same year, compiled a 60-month medical monitoring investigation of 91 Qatari morbidly obese adolescents that underwent LSG surgery between 2011 and 2014, with this study focusing on any potential diabetes management effectiveness demonstrated by LSG surgery [24]. The results of this comprehensive investigation demonstrated that—in addition to psychological benefits, including body image acceptance—LSG surgical treatment served to achieve significant amelioration of short-/long-term obesity-linked co-morbidities, such as total remission of all prediabetic-diagnosed morbidly obese adolescents [24]. Regarding HbA1C serum levels, the presurgical data recorded a mean serum-HbA1C level of 6 mmol/L, which was effectively reduced to 5.1 mmol/L post-LSG surgery (*p* = 0.0001) [24].

Regarding the dataset outcomes from our reported investigation, the main parameters that were analyzed within 64 children co-diagnosed for morbid obesity and type 2 diabetes, and designated for LSG surgery, were HbA1c serum level and random blood sugar analysis. Both parameters were obtained from all patients prior to surgery and after 12 months. The random blood sugar level was found to be reduced by 15.4% (*p* = 0.036) when re-performed post-LSG surgery. In addition, the HbA1c serum level was also reduced by 10.9% within such patients, following the sleeve gastrectomy surgical procedure. Both parameters reflect major biomarkers and biochemical indicators for type 2 diabetes progress and medical management, with both of them being adequately reduced following LSG surgery. These obtained datasets are also in corroboration with the similar studies previously published and described above; thus, this study provided further evidence on the effectiveness of LSG surgery in ameliorating (or even providing total remission) of type 2 diabetes within this patient population, even in childhood-age demographics.

However, several imitations within this study must be mentioned. Firstly, the patient population was relatively constricted, due to the geographical region boundaries on which this study was based upon. Future studies of this nature performed by our group will ensure that a larger patient population will be enrolled within such studies, in order to obtain more statistically reliable dataset outcomes. Secondly, only two main parameters were retrospectively investigated within this study for monitoring LSG effectiveness against type 2 diabetes in such patients, namely HbA1c and RBS analyses. Ideally, additional diabetes-linked biomarkers and biochemical indicators will also be considered for investigation within future studies of this nature performed by our group.

## 5. Conclusions

This study provides further concrete evidence for the beneficial metabolic influence provided by LSG surgery on morbidly obese, childhood-aged patient populations, with effectiveness in reducing co-morbidity progress, in the form of type 2 diabetes, through the reduction in HbA1c levels within the patient’s post-surgery period. However, further and more detailed studies on this research niche are warranted, together with molecular mechanism insights as to how LSG surgery actually triggers such beneficial metabolic influences within these patients.

## Figures and Tables

**Table 1:** Personal data of investigated study participants—a total cohort of 64 children with type 2 diabetes mellitus that underwent LSG surgery were included. Patients’ ages ranged from 5 to 14 years, with a mean age of 11.2 ± 2.3 years old. BMI was 44.63 ± 9 pre-operative and 35 ± 10 post-operative (*p* = 0.001). Overall, 34 (53.1%) children were females and 30 (46.9%) were males.

Personal Data	N	%	
Gender			
Male	30	46.9%	
Female	34	53.1%	
Age in years			
Range	5–14		
Mean ± SD	11.2 ± 2.3		
BMI	Baseline	12 months	*p*-value
Mean ± SD	44.63 ± 9	34.78 ± 9.64	0.001 *

P: Paired t-test. * *p* < 0.05 (significant).

**Table 2:** HbA1c and RBS changes among study participants before and after laparoscopic sleeve gastrectomy.

BGL	Phase						Change%	*p*-Value
	Baseline			12 Months				
	Mean	SD	Median	Mean	SD	Median		
HbA1C	6.0	0.8	5.7	5.4	0.4	5.4	−10.9%	0.001 *
RBS	102.9	34.0	92.7	87.1	17.3	91.0	−15.4%	0.036 *

P: Paired *t*-test. * *p* < 0.05 (significant). % Change: (post value-pre value) * 100/pre value.

**Table 3:** Adjusted and unadjusted means for post-operative HbA1c.

		Unadjusted		Adjusted	
Age	N	M	SD	M	SE
≤11 years old	23	5.32	0.26	5.31	0.26
11–12 years old	20	5.43	0.43	5.42	0.43
>12 years old	21	5.36	0.48	5.36	0.47
**Total**	64	5.40	0.40	5.36	0.40

*N* = Number of participants, M = mean, SD = standard deviation, SE = standard error.

## Data Availability

The datasets used and/or analyzed during the current study are available from the corresponding author on reasonable request.
